# Sexuality and Stroke: The Importance of Considering Cognitive and Perceptual Impairments in Post-Stroke Sexual Functioning

**DOI:** 10.3390/brainsci15080797

**Published:** 2025-07-26

**Authors:** Daniel Geller, Samantha Wong

**Affiliations:** Department of Rehabilitation and Regenerative Medicine, Programs in Occupational Therapy, Columbia University, 617 West 168th Street, New York, NY 10032, USA

**Keywords:** stroke, sexuality, cognition, perception, sexual dysfunction, sexual rehabilitation

## Abstract

Sexuality and intimacy are essential aspects of the human experience for all people, contributing significantly to physical and emotional connections, well-being, and quality of life. Despite their importance, these topics are frequently overlooked in stroke rehabilitation, especially for those with cognitive and perceptual impairments. Existing research on post-stroke sexual rehabilitation tends to focus on sexual dysfunction and the secondary physical and psychological stroke symptoms, with little attention to cognitive and perceptual impairments. Cognitive deficits, such as decreased memory, generalized attention, and executive function not only can hinder sexual participation but also raise the complex issue of capacity to consent. This paper argues that it is imperative for researchers and healthcare practitioners to address cognitive and perceptual challenges, understand consent laws in their respective regions, and consider the influence of culture and social norms in order to support the sexual rights and well-being of all stroke survivors. Furthermore, this article provides some practical recommendations, from an occupational therapy perspective, that healthcare practitioners can provide to clients and their partners.

## 1. Introduction

Globally, there are an estimated 94 million people living post-stroke with an estimated incidence of 12 million new strokes each year. Strokes remain the second leading cause of death worldwide and the third leading cause of disability and death combined [1]. In the United States, there are an estimated 7,000,000 stroke survivors with approximately 795,000 new stroke cases per year, with stroke being the leading cause of serious long-term disability [2]. Post-stroke impairments include physical, cognitive, and/or psychological issues that negatively affect a person’s ability to participate in daily activities and therefore result in a decreased quality of life [3,4]. Sexual activity, an activity of daily living, is often overlooked but is a crucial part of people’s lives and if not addressed has been associated with decreased sexual participation, satisfaction, and quality of life in people with cardiovascular disease [5]. In the past two decades, post-stroke sexual dysfunction research and education primarily focused on physical, psychological, medication-related, and co-morbidity factors [6,7,8,9,10], with very little attention to cognition and perception [5,11,12,13,14,15]. In a 2025 comprehensive literature review, Zhang et al. [16] examined research and reports regarding the prevalence, mechanisms, and treatments related to post-stroke sexual dysfunction. However, post-stroke cognitive and perceptual impairments were minimally addressed, which play a critical factor in sexual participation. Cognition is a conscious process referring to awareness and acquiring knowledge through thought and experiences, while perception is the ability to organize and interpret sensory information in a meaningful way [17]. Post-stroke cognitive impairments occur in up to 70% of stroke survivors and can range from mild to severe impairments [18,19,20] and are associated with decreased occupational performance, quality of life, and caregiver outcomes [21,22]. Thus, it is the authors opinion that it is crucial for healthcare practitioners and researchers to examine cognitive and perceptual impairments post-stroke as they are related to sexual participation and quality of life in the context of culture, religion, and social norms.

## 2. Sexuality, Intimacy and Sexual Health

Sexual activity is the engagement of sexual acts with oneself and/or with others and can include, but is not limited to, oral, vaginal, and anal sex; touching, caressing, and licking; use of sex toys and/or sex videos; and mutual masturbation [23]. Sexuality is an integral part of being human, spanning across one’s life, and involves more than just sexual activity and procreation but also gender roles and identities, sexual orientation, pleasure, and eroticism [24]. Intimacy is defined as a close and personal relationship with another person, which involves trust, support, and affection and does not have to be of a sexual nature. When working with post-stroke clients, it is important to consider their sexuality, desires and interests, sexual orientation, and gender identity, as well as their culture, religion, and social context [25]. According to the World Health Organization [24], sexual health refers to a state of sexual well-being, encompassing more than just disease or sexual dysfunction. It involves a positive and respectful approach to sexual and intimate relationships, free of coercion and violence. Furthermore, sexual health is also considered a human right and should be accessible to all people including stroke survivors with cognitive and perceptual impairments. However, research conveys that many people have a decline in sexual frequency and satisfaction post-stroke [7,8,10,11,26,27,28]. Vikan et al. [29] examined sexual satisfaction among stroke patients in cognitive rehabilitation and found that only 33% were satisfied with their sex lives. This dissatisfaction was associated with decreased sexual activity, increased anxiety, a fear of partner rejection, and sleep disturbances. Studies have also shown that individuals post-stroke are interested in receiving information about sexual activity, yet few report this need being met [30,31,32]. Auger et al. [30] examined the sexual rehabilitation needs of individuals in the subacute phase of stroke recovery at a Canadian rehabilitation hospital. Although clients reported sexuality as a secondary priority after basic activities of daily living (ADL), they all agreed on the importance of at least discussing sexual and intimacy participation. However, the study findings are difficult to generalize to the general stroke population due to the small sample size of five. Prior et al. [31] examined the gaps in post-stroke sexual activity amongst 1265 rehabilitation participants in Australia, which indicated that 30% desired information about sexual activity, whereas only 8.2% received it. The authors argue that this further emphasizes the importance and missed opportunity of providing sexual rehabilitation for people with cognitive and perceptual impairments post-stroke.

## 3. Stroke and Sexual Dysfunction

There is a common saying that the largest sex organ is the brain, which highlights the profound influence of the brain on sexual arousal, experiences, and pleasure. Therefore, it is crucial for healthcare practitioners to have knowledge of the brain and sexual function in relation to a stroke. A stroke occurs when there is a lack of blood flow and oxygen to an area of the brain, which leads to brain cell death [4]. As a result of a stroke, individuals can have an array of symptoms, including sexual dysfunction, which is dependent on both stroke location and laterality [6,7,9]. In Contrada et al. [6], the authors examined the neurological mechanism associated with sexual dysfunction. Brain lesions in the medial frontal cortex, anterior cingulate cortex, thalamus, hypothalamus, insula, and claustrum were related to erectile dysfunction. Hyposexuality was related to lesions in the medial frontal cortex, while hypersexuality was related to lesions in the temporal cortex and bilateral damage to the amygdala. Right parietal lesions were associated with altered cognitive arousal or neglect, while cerebellar lesions were associated with ejaculatory disorders, anhedonia, and altered emotional arousal. Regarding stroke laterality and sexual dysfunction, there is a lack of consensus on its association [7]; however, it has been speculated that the right hemisphere, which is dominant for attention, is more likely to play a role in the activation and attention of libido and erectile function, as well as in the recognition of emotional stimuli [9].

## 4. Sexual Rehabilitation

In a 2025, Zhang et al. [16] examined the prevalence, clinical features, and interventions for people with post-stroke sexual dysfunction. This review included 20 studies that highlighted a high prevalence of sexual dysfunction, as well as the negative effects of secondary factors on sexual participation. These secondary effects included the following: (1) psychological problems, such as depression, anxiety, and low self-esteem; (2) physical impairments, such as hemiplegia, spasticity, facial droop, dysarthria, incontinence, and decreased sensation; (3) vascular factors such as hypertension, diabetes, obesity, and smoking; and (4) medication side effects, such antihypertensive or antidepressant medications that can cause decreased libido. The intervention approaches discussed in this review focused on sexual counseling pertaining to psychological and sexual dysfunction, medical and physical interventions such as medication changes, and pelvic floor training, respectively. However, it is noteworthy that the majority of the studies in this review were single -center studies with weak methodological designs and small sample sizes [16]. Auger et al. [33] performed a systematic review of sexual rehabilitation interventions from allied health professionals after stroke. Eight studies from various countries, including South Korea, Australia, India, United States, Denmark, and Canada, focused on physical and psychological interventions, such as pelvic floor therapy and counseling. Additionally, one study addressed the needs for individuals with aphasia. The results showed that interdisciplinary sexual rehabilitation improved sexual functioning and satisfaction. Two of the studies, however, had lower quality methodological designs, which creates potential bias and affects the overall quality of the review. While the authors of both studies [16,33] acknowledged the lack of high-quality research in post-stroke sexual dysfunction, neither discussed the role of cognitive impairment or the gap in the literature in the context of their impact on sexual participation. Notably, the search strategies in both studies included stroke-, sexuality-, and sexual dysfunction-related terms but included nothing related to cognition or perception. However, the authors of this study performed a literature review with these key terms with limited relevant findings. In a quasi-experimental study design, Song et al. [15] examined sexual rehabilitation interventions for stroke patients and their spouses. The intervention focused on general sexual health education, sexual life changes after stroke, physical impairment, and counseling for fear of a sexual life post-stroke, with minimal emphasis on cognitive and perceptual deficit treatments. The results demonstrated improvements in sexual satisfaction and sexual activity but not sexual knowledge. While the above-mentioned secondary factors associated with stroke are important to consider and have promising interventions, cognition and perception were minimally addressed, despite their critical role in supporting meaningful sexual participation.

## 5. Cognitive/Perceptual Impairments

Cognitive impairment refers to the decline in the thinking and thought processes needed to acquire or maintain knowledge and includes, and is not limited to, memory, generalized attention, executive functioning, and initiation. Perceptual impairments refer to the inability to organize and interpret sensory information leading to challenges interacting with the world. These impairments include, but are not limited to, motor apraxia, neglect (i.e., unilateral attention deficit), spatial relations dysfunction, and perseveration [17]. Both cognitive and perceptual impairments can lead to challenges in daily activities such as sexual activity.

Stroke location, severity, and clinical presentation must also be considered as they pertain to cognitive and perceptual impairments, as stroke location directly influences the specific type of cognitive impairment and symptoms. For instance, a person who had a stroke in the prefrontal area may have difficulty with executive functioning, while a person with a stroke in the limbic or orbital frontal area may have memory issues. Based on severity, a post-stroke person may have mild, moderate, or severe impairments, as well as a single or multiple cognitive impairments. In addition, cognitive impairments have different sub types, with each presenting differently. For instance, memory impairments can be short or long-term deficits, episodic or semantic or procedural deficits which each requiring different treatment approaches. Additionally, it is also important to note that these impairments can impact people in different ways. For example, two people with minimal left neglect may have difficulty with different tasks and activities [4,17]. In a systematic review and meta-analysis, Stolwyk et al. [22] demonstrated that impairments in both cognition and perception in post-stroke individuals were associated with limitations in activities of daily living (e.g., dressing, bathing), instrumental activities of daily living (e.g., meal preparation), and overall participation restrictions. Thus, the authors, who are occupational therapy (OT) practitioners, extrapolated that cognitive and perceptual impairments would be associated with limitations in sexual activity as per the Occupational Therapy Practice Framework: Domain and Process Fourth Edition. This document describes occupational therapy practice and categorizes sexual activity as an ADL [34]. Therefore, the authors argue that it is crucial for healthcare practitioners to perform a thorough medical history and cognitive evaluation as it pertains to ADLs including sexual activity.

While cognitive and perceptual impairments can negatively impact post-stroke sexual functioning, there is limited education and research in this area. For example, in a study examining post-stroke sexual dysfunction in men, Calabrò [35] mentions neglect, apraxia, and executive functioning, however, does not expand on these topics. In addition, in two nursing articles with a focus on practical strategies for post-stroke individuals, Kautz and Horn [12] discuss concrete thinking and Kautz [13] discusses cognition, consent, and memory impairments related to sexual activity; however, both articles focus on the physical and psychological issues. Furthermore, Seymor and Wolf [14] studied sexual participation after a mild stroke and found a negative correlation between sexual dysfunction and memory (i.e., increased sexual dysfunction associated with decreased memory), however this was not the focus of the study. Therefore, the authors emphasize the importance of addressing cognitive and perceptual impairments in sexual functioning among post-stroke individuals in both research and clinical practice.

### 5.1. Consent

Initially, one important consideration when working with individuals with post-stroke cognitive, perceptual, or language impairments is their capacity to consent to sexual or intimate activities [13]. Research has shown that people with cognitive and/or perceptual impairments are more vulnerable to financial or sexual exploitation [36]. In the United States, the legal definition of consent is based on three criteria: knowledge, intelligence, and voluntary agreement to participate in sexual activities. The knowledge component entails, but is not limited to, the individual’s ability to recognize their partner(s) in the relationship, understand basic sexual anatomy, and awareness of the relevant facts associated with the sexual activity. The intelligence component involves the ability to understand the risks and benefits of engaging in the sexual activity, such as possibility of pregnancy or experiences of pleasure. Finally, voluntariness refers to the person’s ability to recognize situations involving sexual abuse or coercion and to act accordingly [37]. However, each state may interpret these criteria differently or may have added criteria. For example, in 13 U.S. states, courts require individuals to understand the potential consequences of sexual activity, such as contracting sexually transmitted infections. Meanwhile, in nine states, courts consider the presence of a disability as a factor that may impact the ability to provide consent [38]. While the discussion of consent has focused on the U.S., other countries have different laws. Many European countries rely on coercion-based rape laws, which focus on the perpetrator and their use of force and coercion prior to the sexual act, with little regard to the victim and their consent [39]. However, there has been a shift towards consent-based rape laws, which prioritize the absence of the victims’ consent and protect the victims’ sexual autonomy. According to Amnesty International, in 2023, the Netherlands became the 17th European state out of 31 to adopt consent-based laws [40].

Interestingly, in the U.S., seven states require an understanding of the moral quality of engaging in sexual activity as a part of consent [38]. However, the concept of the moral quality of sexual activity is subjective and differs according to the country, culture, religion, and social norms. For instance, the Catholic Church traditionally views sexual activity primarily as a means for procreation within marriage, often portraying it in a restrictive or negative light. In contrast, Judaism views sexuality more positively, as a natural means for procreation but also as an expression of love and intimacy. Buddhism is centered around following an ethical life focusing on norms of morality and behaviors such as avoiding adultery, refraining from sexually exploiting vulnerable populations, and abstaining from sexual abuse. It should be noted that religious groups have a range of beliefs that may be more conservative or liberal in nature [41]. Culture also plays a role in moral quality. For instance, Collier et al. [42] examined the attitudes of adolescents in the U.S. and the Netherlands towards gays and lesbians. The results showed that the Dutch were more accepting but more interestingly, American participants justified their attitudes based on social norms and religious opposition, while the Dutch participants relied on individual rights and the genetic basis of homosexuality.

Ultimately, regardless of cultural or moral frameworks, the central question remains whether the person with a cognitive, perceptual, or language deficit has the capacity to consent. It should be noted that only certain healthcare professionals are able to determine capacity, such as mental health professionals, physicians, or legal experts. While allied health professionals, such as occupational therapists, physical therapists, nurses, and speech therapists, may not be able to determine capacity for consent, they still have a role in the person’s capacity to consent through education focused on the required criteria by state or country. For instance, this education may entail teaching the body parts, consequences of sexual activity, and the appropriate context for sexual activity [37]. Thus, the authors stress the importance of understanding consent laws in one’s area but also considering culture, social norms, and religion.

### 5.2. Cognitive and Perceptual Impairment and Sexual Participation

While consent is critical to address with cognitively impaired stroke survivors, other cognitive and perceptual impairments can affect sexual participation (Table 1). For example, a person with memory problems may forget the steps of the sexual activity (e.g., preplanning contraception or using sex paraphernalia), forget that the activity was just performed, or perseverate on sexual activity, thus pressuring the partner for sex [13]. A person with impaired executive functioning may have difficulty initiating sex, organizing and/or planning an intimate or sexual activity, or have difficulty following directions. A person with impaired attention and concentration may be easily distracted or unable to focus on the activity at hand. Regarding perceptual impairments, a person with neglect may not be able to attend to one side of their body, which may hinder sexual activity or intimacy. A person with motor apraxia may have difficulty with coordinating hand and arm movements for sexual activities such as oral sex [17,43]. Thus, cognitive and perceptual impairments can impede one’s ability to participate in sexual activity. Therefore, these impairments should be addressed during sexual rehabilitation.

### 5.3. Interventions and Recommendations

Functional cognition involves the integration of cognitive abilities, such as attention, memory, and executive functioning, and applying them to real-world activities and situations [44]. In other words, it refers to how people use their thinking and processing skills to perform daily activities, such as sex. Occupational therapy practitioners, experts in functional cognitive rehabilitation, activity analysis, and the therapeutic use of meaningful occupations, play a vital role in sexual rehabilitation, especially for stroke survivors with cognitive impairments. The following are general recommendations, from an OT perspective, for healthcare practitioners working with stroke survivors and their partner(s). First, practicing cultural humility, which is understanding one’s own culture and how it affects their interactions with people of different cultures, is critical in building trust between the healthcare practitioner and the client. More specifically for sexuality, it is important to reflect on the concept of humans as sexual beings and to explore one’s own sexual self. Sexual self refers to how one views their own sexual being, including but not limited to, their sexual preference, sexual expression, and gender identity, as well as one’s self-worth and body image. Some strategies for sexual self-reflection are reframing, which is a cognitive strategy whereby one consciously thinks about alternative responses to a negative experience or journaling whereby one can not only slow down their thought process through writing but also reflect and be free with one’s thoughts [25]. Second, open communication, respect, and mutual understanding is important for all relationships but especially relationships with people with cognitive impairments [43]. This can be accomplished by determining the best communication strategy between partners, as it is of vital importance pertaining to consent and a safe and satisfying sexual and intimate encounter. These communication strategies can be verbal, tactile, gestural, or auditory in nature and will differ depending on the person’s cognitive impairments and the relationship between the partners. Third, all sexual rehabilitation interventions should be client-centered; therefore, treatment should be implemented according to the client’s culture and social structures, as well as their sexual activities, interests, and expressions [45]. Thus, the authors argue it is imperative to not only perform a comprehensive stroke evaluation but also a sexual history to establish client-centered goals around sexuality and intimacy.

#### Specific Interventions

This section provides specific recommendations and strategies for both the client and their partner(s), based on impairment type. It should be noted that this paper is not intended to provide an in-depth review of each impairment type and a comprehensive intervention approach; rather, it provides some basic strategies and encourages readers to seek out other resources or develop research initiatives in this area. Furthermore, these recommendations are based on previous research on the effectiveness of interventions for clients with cognitive or perceptual deficits post-stroke [46] and OT textbooks [47,48], and were then adapted for sexual activity and participation, as needed.

Due to clients’ differing cognitive severities and recovery trajectories, healthcare practitioners should use their clinical judgment while implementing treatment interventions. For instance, a client with a large stroke who presents with severe neglect may require hand-over-hand strategies to incorporate the neglected limb into the sexual activity. A client with moderate neglect may require several verbal cues, whereas a client with mild neglect may need only a few verbal cues to incorporate the limb. The recovery trajectory also needs to be considered as a client with a good prognosis may improve, thus requiring less assistance, meanwhile a client with a poor prognosis may need continued assistance to participate in sexual activity [47].

A client with generalized attention and concentration impairments may not be able to participate in sexual activities due to being easily distracted or having difficulty maintaining focus on the sexual activity at hand. Thus, the following are possible treatment approaches: (1) decrease distractions in the environment such as eliminating clutter in the room, finding a quiet room, closing doors and curtains, keeping phones turned off, and facing away from distractions, or (2) simplify the sexual activity or engage in one sexual activity at a time. For instance, mutual oral sex may be too difficult as the client must attend to themselves and their partner, therefore one at a time may be more suitable. (3) Plan a specific time of the day without time constraints when the person is the most attentive, and (4) educate the partner(s) to guide the activity through direct verbal cues, gestures, or hand-over-hand assistance, as needed [47].

A client with memory impairments may have difficulties in remembering specific sexual activities or that it just occurred. Thus, the following are possible treatment approaches: (1) use pictures and/or videos of sexual activities for memory recall, (2) simplify the environment by removing clutter, (3) keep a diary to organize thoughts and strategies that work, (4) keep a planner to schedule sexual activity and intimacy, (5) keep all sex paraphernalia such as sex toys, bolsters, lubrication, and condoms organized in a specific place, and (6) educate the partner(s) to guide the activity through direct verbal cues, gestures, or hand-over-hand assistance, as needed [13,46,47].

A client with executive dysfunction may have difficulty initiating, sequencing, planning, and organizing sexual activities or with participation. Thus, the following are possible treatment approaches: (1) organize the room and sex paraphernalia, (2) keep a checklist to establish a sexual activity routine, (3) keep a diary for planning sexual activities, (4) use sequencing cards with sexual positions, (5) simplify the sexual activity or engage in one sexual activity at a time, and (6) educate the partner(s) to guide the activity through direct verbal cues, gestures, or hand-over-hand assistance, as needed [47].

A client with neglect, which is the failure to attend to one side of the body (i.e., affected side) that is not attributed to motor or sensory impairments [17], may have difficulty incorporating the neglected side of the body into the sexual activity or finding objects on the neglected side. Thus, the following are possible treatment approaches: (1) safe and proper positioning of the neglected side to prevent pain, skin breakdown, or uncomfortable positions, and (2) educate the partner(s) to guide the activity through verbal cues, gestures, or hand-over-hand assistance for the incorporation of the stroke survivor’s affected limb, as needed. For clients with minimal-to-moderate neglect, educate the client and partner on increasing their awareness of the affected side by positioning the partner on the affected side, massage and/or use of lotion, undressing/dressing the neglected limb, vibration, kissing, licking, or physically moving the neglected side for a whole-body experience. For a person with severe neglect, compensatory strategies may be preferable such as placing sex toys, condoms, lube, or the partner(s) on the unaffected side, thus making it easier for the stroke survivor to attend to the person or objects needed [46,47,48].

A client with motor apraxia, which is a dysfunction of motor movement and coordination not attributed to motor or sensory impairments [17], may have difficulty coordinating the arm and hand during sexual activities. The following are possible treatment strategies [46,47]: (1) educate the partner(s) to perform hand-over-hand assistance or the stroke survivor using bilateral hands during oral or manual sex, (2) use of sex toys that require less arm/hand coordination, such as vibrators and automatic male masturbators, (3) hand-free sex toy mounts, wedges, and bolsters, and (4) have stroke survivors move the arm/hand across supported surfaces to provide maximal sensory feedback (i.e., move hands across chest and stomach to get to the penis, vulva, or anus).

## 6. Conclusions

Sexual health is a fundamental human right and individuals, regardless of their disability, deserve the opportunity to engage in fulfilling sexual and intimate relationships. However, post-stroke sexual rehabilitation research and practice have primarily focused on physical and psychological consequences, leaving a gap in the understanding and treatment of cognitive and perceptual impairments. To promote holistic care and recovery, the authors argue that it is essential for researchers and healthcare practitioners to focus on cognitive and perceptual impairments in relation to sexual participation. This includes, but is not limited to, exploring the role of cognitive and perceptual impairments and sexual participation, examining the complexity of sexual consent, evaluating the effectiveness of interventions, and ensuring the inclusion of diverse populations, particularly gender and ethnic minorities, whose experiences are often underrepresented. A more comprehensive, inclusive, culturally sensitive, and evidence-based approach is necessary to support the sexual rights for all stroke survivors and their partners.

## Figures and Tables

**Table 1:** Cognitive/perceptual impairments and sexual participation.

Cognitive/Perceptual Impairment	Effects on Sexual Activity with Partner(s) or Self
Attention and concentration	Difficulty with the following: Attending to the sexual activity Changing from one sexual activity to another Maintaining attention during the sexual activity Attending to both giving and receiving pleasure at the same time Attending to the partner(s) needs
Executive function	Difficulty with the following: Organization and/or planning for sexual activity Decision making about positions, sexual activity, or location Problem solving when sexual activity is not working Initiating sexual activity Following or giving directions during sexual activity
Memory	Difficulty remembering the following: The steps of the sexual activity Contraception or use of sex paraphernalia One just had sexual activity The different kinds of sexual activities or positions Their own or partner’s likes and dislikes
Neglect	Difficulty with the following: Attending to one side of their body (e.g., arm, leg, or breasts) Attending to the spatial area of one side of their body Finding sexual paraphernalia on one side of the bed
Motor apraxia	Difficulty coordinating arm/hand movements: During specific sexual activities with partner (oral or manual sex) During masturbation During sensual touch and/or massage With use of sex paraphernalia To change positions during sexual activity

## Data Availability

No new data were created or analyzed in this study.
